# Modifying the Mini-Cog to Screen for Cognitive Impairment in Nonliterate Individuals

**DOI:** 10.1155/2021/5510093

**Published:** 2021-08-16

**Authors:** Shambhu P. Adhikari, Rubee Dev, Soo Borson

**Affiliations:** ^1^School of Health and Exercise Sciences, University of British Columbia, Kelowna, British Columbia, Canada; ^2^Faculty of Nursing, University of Alberta, Edmonton, Canada; ^3^Department of Psychiatry and Behavioral Sciences, University of Washington, Seattle, USA

## Abstract

**Objectives:**

The Mini-Cog, a rapid, valid, and reliable screening tool for cognitive impairment, consists of 3-word recall and an executive clock drawing test (CDT). However, CDT requires at least basic literacy and cultural exposure to analog clocks, conditions not met in many population groups around the world. We developed a modification of the Mini-Cog (MMC) for use with nonliterate and literate individuals.

**Methods:**

Participants were adults (≥60 years) with no neurological diagnosis, with known cognitive impairment due to stroke, Parkinsonism, traumatic brain injury, or Alzheimer's disease, and whose family members were able to read and write. We replaced the CDT with two tasks of everyday life: a serial subtraction task or a multistep performance task. Family members rated the acceptability and feasibility of the Mini-Cog versions using a 6-point scale and completed a proxy-rated cognitive staging tool, the Dementia Severity Rating Scale (DSRS). Spearman's rho, Mann-Whitney *U*, and chi-square tests were used to evaluate group differences and associations between measures.

**Results:**

Data were collected from 63 participants (75% ≥ 60 years, 67% nonliterate). Literacy was associated with CDT (chi-square strength 0.9, *p* < 0.001). Both MMC versions correlated with DSRS in healthy adults and patients (rho 0.6-0.7, *p* < 0.05). In literate individuals, the acceptability and feasibility of CDT and both alternate distractors were similarly high (5/6).

**Conclusions:**

Two alternate distractor tasks may successfully replace CDT in the Mini-Cog. The MMC versions are promising and deserve further study as screening tools for cognitive impairment in larger and more fully characterized samples.

## 1. Introduction

The Mini-Cog (MC) is a short screening tool for clinically important cognitive impairment, originally developed by Borson et al. in 2000 by combining a short memory test and an executive clock drawing test (CDT) [1]. The choice of 3-item recall as the memory test was based on research showing that this element in the Mini-Mental State Examination (MMSE) is the first to be impaired in early Alzheimer's disease [1, 2]. The CDT serves as an informative distractor: it creates a short recall delay and itself functions as a cognitive test, since it relies on multiple integrated cognitive functions commonly impaired in neurocognitive disorders. The MC is feasible, reliable, valid, and time saving for settings in which rapid detection of cognitive impairment is desirable, since it takes just 3 minutes to administer [1, 3]. It has acceptable sensitivity and specificity for dementia in both clinical and population samples, detects mild cognitive impairment (with less sensitivity and specificity), and has been widely implemented in western clinical settings [1, 2, 4]. McCarten and colleagues highlighted its speed and high acceptance by older veterans [5]. Studies have also reported superior screening properties of the MC compared to that of MMSE [1, 6], and its psychometric properties have been well established [1, 7]. Both components of the MC contribute to the detection of dementia [2].

The Mini-Cog is available in many languages (http://mini-cog.com) and has been used successfully in the United States with both English and non-English speakers with varying levels of education, but its clock drawing component is vulnerable to very low education—uncommon in US-born populations and poorly represented in most research on cognitive impairment. The CDT may not be valid with nonliterate individuals, as it generally requires at least a basic education (typically 4-5 years of primary schooling); while oral 3-word recall should not be influenced by literacy, clock drawing errors are common and often different in character among individuals with fewer than 5 years of education, obscuring its discriminative power for acquired cognitive impairment [8]. To make a MC-like tool feasible for rapid screening of nonliterate individuals for cognitive impairment, the CDT must be replaced by another task that serves a similar function. Other screening tests such as the MMSE have been modified for different levels of education [9] but not for nonliterate individuals. In the Bangla version of the MMSE, validated in both literate and nonliterate groups, Kabir and Herlitz replaced its calculation item with a real-life mathematical calculation task used every day by both literate and nonliterate individuals [10]. This type of real-life task could also potentially replace the CDT, but because a small percentage of nonliterate individuals may not be able to do it, we introduced a multistep matching performance task as another option, reasoning from the multistep command used in the MMSE [9]. We created two modified versions of the Mini-Cog (MMC), one using an everyday serial subtraction task (SST) and the other a multistep performance task (MPT) in place of CDT (Appendix 1).

## 2. Materials and Methods

### 2.1. Setting and Participants

**Figure 1:**
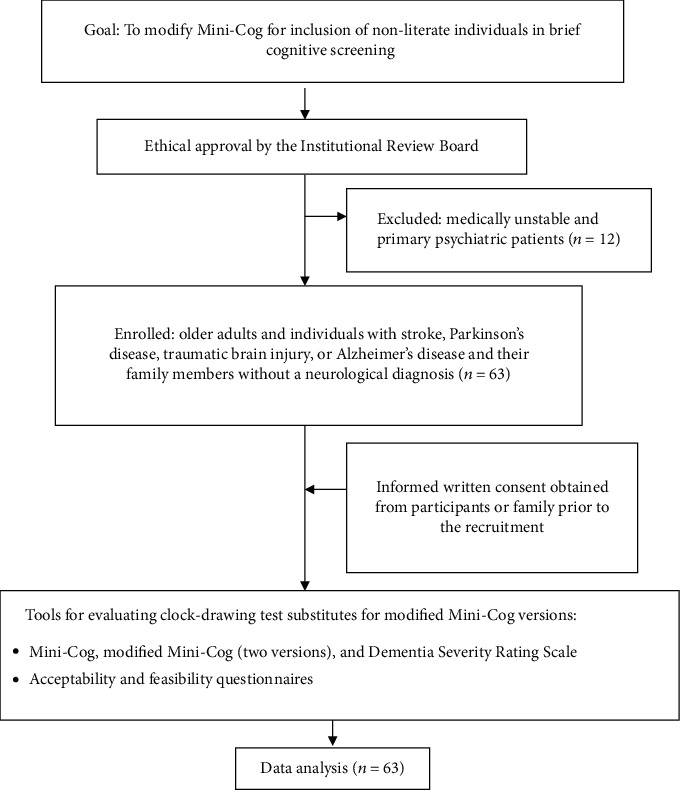
Procedural flowchart.

Nepali-speaking individuals were recruited among patients and family members seen in a community hospital in Nepal during April to December 2019. Purposive sampling was used to identify potential participants for screening test comparisons. Inclusion criteria generated two groups: older adults (≥60 years) with no diagnosis of neurological disease and individuals of any age with diagnosis of a neurological disease (stroke, Parkinson's disease, traumatic brain injury, or Alzheimer's disease) based on medical records. All had to be able to understand Nepali language and respond orally to interview questions and have a participating family member who could read and write. Unconscious or medically unstable patients and individuals with confirmed primary psychiatric illness were excluded. The procedural framework is presented in Figure 1.

Literacy was defined pragmatically for this project. Participants were first asked if they were able to write and read (yes/no) and then to read a word from the 3-word registration task. They were then offered paper and pencil and invited to write anything they liked. Those answering “no” and demonstrably unable to read or write were classified as nonliterate and presumed unable to do clock drawing.

### 2.2. Measures

#### 2.2.1. Mini-Cog Versions

Literate participants completed the original MC and both versions of MMC in random order. Nonliterate participants were not asked to do the MC, which requires drawing a clock, but completed both versions of MMC.

#### 2.2.2. Measure Feasibility and Acceptability using Questionnaires

Participants rated each component of the MC and the two MMC versions (3-word registration, 3 questions; each distractor, 3 questions) using a 6-point scale developed for this study (Appendix 2). Each question was scored yes (1 point) or no (0 point). The total score was calculated by adding the score of the registration component and the score of one distractor for a total score of 0-6 for each of the three test versions. Higher scores indicate better acceptability and feasibility.

#### 2.2.3. Preliminary Validation of Modified Mini-Cog Versions using the Dementia Severity Rating Scale

Validation of a new or modified screening test requires comparison with an external standard. For nonliterate as well as literate individuals, we used a structured proxy interview, the Dementia Severity Rating Scale (DSRS). The DSRS, based on the Clinical Dementia Rating, taps everyday cognitive functioning based on observations of a family member or another individual who knows the person well [11, 12]. Its psychometric properties have been well established in studies from the United States (interrater reliability = 0.9, intrarater reliability = 0.8, internal consistency reliability > 0.7, and validity = good [11–13]). Scores can range from 0 to 54 (normal cognition to severe dementia, higher scores indicating greater impairment).

The Mini-Cog versions (original as well as modified versions) and the DSRS all were administered on the same day for an individual by a single assessor.

### 2.3. Statistical Analysis

Demographic variables were analyzed using descriptive statistics. The chi-square test was used to examine the association between the variables. Concurrent validity was evaluated comparing MMC versions with DSRS. Phi or Cramer's *V* was used (as the data in the Mini-Cog are analyzed in the form of categorical scale [1]) to report strength of association for a 2 × 2 contingency table and bigger than 2 × 2 tabulation, respectively [14]. The Mann-Whitney *U* test was used to evaluate group differences. The significance level was set at *p* < 0.05. Data were analyzed using SPSS version 21.0.

## 3. Results

Out of 75 participants screened, data were collected from 63 participants (12 participants were excluded because they were medically unstable and/or had primary psychiatric diagnosis). Out of 63 participants, about 71% (*n* = 45) were adults with no neurological diagnosis and about 29% (*n* = 18) were individuals with a neurological diagnosis. Two-thirds of the participants (*n* = 42) were nonliterate (unable to read or write at all). Demographic and clinical characteristics of participants are presented in Table 1.

In individuals without neurological disorder having a word recall score of 3/3 (*n* = 28; 11 literate, 17 nonliterate), literacy was strongly associated with self-reported understanding of the CDT (*χ*^2^: 20.5, *p* < 0.001, strength of association: 0.9). There was no significant difference between literate and nonliterate groups on the SST (*n* = 28, Mann-Whitney *U*: 66.5, *p* = 0.13) or the MPT (*n* = 28, Mann-Whitney *U*: 77.5, *p* = 0.34).

The association between different Mini-Cog versions is shown in Table 2.The DSRS was significantly correlated with all Mini-Cog versions (Phi or Cramer's *V* ranged from 0.7 to 0.9, *p* < 0.05) in literate as well as nonliterate participants. Both versions of MMC were significantly associated with MC (Phi or Cramer's *V*: 0.7, *p* = 0.003) in literate participants. Two versions of MMC were also significantly associated with each other (Phi or Cramer's *V* ranged from 0.6 to 1.0, *p* < 0.05) separately in different groups; literate, nonliterate, those with no neurological diagnosis, those with neurological diagnosis, and total participants.

**Figure 2:**
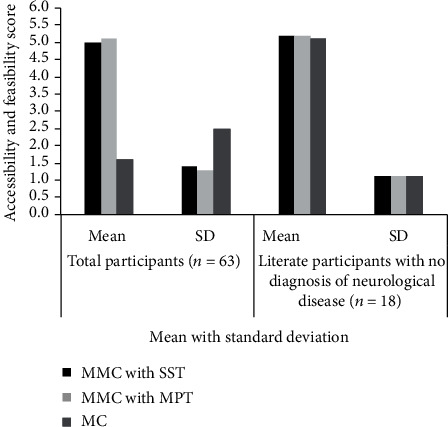
Accessibility and feasibility of different Mini-Cog versions. MC: Mini-Cog; MMC: modified Mini-Cog; SST: serial subtraction task; MPT: multistep performance task; SD: standard deviation; *n*: sample size.

Scores for acceptability and feasibility of different Mini-Cog versions are shown in Figure 2. In literate participants without neurological disease, scores for all three versions were essentially identical. When all participants (nonliterate, literate, and those with or without neurological diagnosis) were included in the analysis, mean and standard deviations of acceptability and feasibility scores for the two MMC versions were similar to each other and to scores for literate subjects, but much lower (and standard deviation much higher) for the original MC owing to the inability of nonliterate participants to do the CDT.

## 4. Discussion

Individuals lacking basic literacy are difficult to screen for cognitive impairment. Our study is unusual in its effort to retain the brevity and simple scoring of the Mini-Cog, a brief cognitive screening tool, while replacing its literacy-sensitive clock drawing task [8] with everyday complex cognitive tasks familiar to both literate and nonliterate individuals. The two resulting alternate versions of the Mini-Cog, SST and MPT, correlated significantly and similarly with the DSRS, a proxy-rated cognitive impairment screening tool. Both nonliterate and literate individuals rated both the SST and MPT applicable, acceptable, and feasible.

The absence of significant group differences between literate and nonliterate individuals in SST or MPT score, and their significant association with the DSRS as rated by family informants, provides preliminary evidence that both modified versions of the Mini-Cog may be acceptable screening tests applicable to both literate and nonliterate individuals. While the SST and MPT lack the visual performance record of the original Mini-Cog, the scoring was set to replicate the binary method established for the CDT in the original Mini-Cog [1]. Instructions for administering both modified versions are simple; reducing the chance of spurious poor performance due to inadequate test administration is low. Furthermore, because there are two distractors, either one can be selected based on participants' choice and ability. If an individual fails one, a second distractor can be tried. This is in line with the administration strategies of MMSE [9]. However, equivalent performance of the two versions cannot be inferred from this proof-of-concept study.

The validity of the two MMC versions as screens for cognitive impairment was evaluated by comparing results with the informant-rated DSRS; correlations were large [14] in nonliterate groups with and without neurological disease. The SST and MPT were strongly correlated with each other, the CDT, and the DSRS in literate participants who could perform all three MC versions. Results demonstrated high acceptability and feasibility for both CDT substitutes in the sample as a whole.

## 5. Limitations

As a proof-of-concept study, our sample size is relatively small and no independent, fully objective assessment of cognition was possible. However, the association of scores with the DSRS—an acceptable method for classifying cognitive function based on informant observations—is a reasonable place to start [11] in settings and populations very different from those for which cognitive tests are typically created, including developing countries where the illiteracy rate of the older population is high [15]. Although our initial findings are promising, they cannot establish the true equivalence of the MMC versions. There might be a selection bias when we recruited only those participants whose family members were literate. However, the situation having every family member as nonliterate is a rare condition.

## 6. Conclusion

Two alternate distractor tasks may successfully replace clock drawing in the Mini-Cog and improve its validity in nonliterate populations and facilitate meaningful population comparisons. The two MMC versions are promising and deserve further study as screening tools for cognitive impairment in larger, more fully characterized samples of mixed literacy, language, and cultural and clinical status.

## Figures and Tables

**Table 1:** Demographic and clinical characteristics of the participants.

Variables	Mean (SD)/*N* (%)			
	Participants with no neurological diagnosis	Participants with neurological diagnosis	All participants	Literate participants
Sample size (*n*)	45	18	63	21
Age (years)	68.3 (6.9)	59.9 (16.7)	65.9 (11.2)	65.1 (11.3)
Gender				
Male	26 (57.8%)	8 (44.4%)	34 (54.0%)	16 (76.2%)
Female	19 (42.2%)	10 (55.6%)	29 (46.0%)	5 (23.8%)
Literacy				
Literate	18 (40.0%)	3 (16.7%)	21 (33.3%)	21 (100%)
Nonliterate	27 (60.0%)	15 (83.7%)	42 (66.7%)	—
DSRS score	6.5 (9.4)	11.0 (8.8)	7.8 (9.4)	6.8 (9.9)
MMC with SST	3.5 (1.4)	3.0 (1.5)	3.4 (1.5)	—
MMC with MPT	3.4 (1.4)	3.0 (1.6)	3.3 (1.5)	—
MC	—	—	—	3.6 (1.5)

MC: Mini-Cog; MMC: modified Mini-Cog; SST: serial subtraction task; MPT: multistep performance task; DSRS: Dementia Severity Rating Scale. Neurological diagnosis: stroke = 13 (20.6%), traumatic brain injury = 4 (6.3%), and Alzheimer′s disease = 1 (1.6%). Out of 21 literate participants, those with no diagnosis of neurological disease were 18 (85.7%) and those with diagnosis of neurological disease (stroke: 2, traumatic brain injury: 1) were 3 (14.3%). There were no significant differences (*p* > 0.05) on any variables between two (with neurological and without neurological) groups.

**Table 2:** Association between different Mini-Cog versions and with DSRS.

Variables		Literate participants (*n* = 21)			Nonliterate participants (*n* = 42)				Total participants (*n* = 63)	
		MMC with MPT	MMC with SST	MC	With no neurological diagnosis (*n* = 27)		With neurological diagnosis (*n* = 15)		MMC with MPT	MMC with SST
					MMC with MPT	MMC with SST	MMC with MPT	MMC with SST		
MC	*χ* ^2^	48.4	42.0							
	*p* value	0.003^∗^	0.003^∗^	—	—	—	—	—	—	—
	Phi/Cramer's *V*	0.7	0.7							
MMC with SST	*χ* ^2^	42.4			31.1		10.3		63.8	
	*p* value	0.002^∗^	—	—	0.03^∗^	—	0.01^∗^	—	<0.001^∗^	—
	Phi/Cramer's *V*	0.7			0.6		0.8		1.0	
DSRS	*χ* ^2^	73.1	69.1	81.5	66.1	90.2	44.7	32.9	139.8	149.5
	*p* value	0.01^∗^	0.003^∗^	0.003^∗^	0.04^∗^	0.007^∗^	0.02^∗^	0.04^∗^	0.002^∗^	<0.001^∗^
	Phi/Cramer's *V*	0.8	0.9	0.9	0.8	0.8	0.8	0.8	0.7	0.7

^∗^*p* < 0.05. *χ*^2^: chi-square; MC: Mini-Cog; MMC: modified Mini-Cog; SST: serial subtraction task; MPT: multistep performance task; CDT: clock drawing test; DSRS: Dementia Severity Rating Scale. All distractors were scored either 2 (correct) or 0 (incorrect). The CDT scoring in MC followed the rules empirically developed for the standardized version of the Mini-Cog (http://mini-cog.com).

## Data Availability

The datasets generated and/or analyzed during the current study are available from the corresponding author on reasonable request.

## Abbreviations

MC: Mini-Cog
MMC: Modified Mini-Cog
CDT: Clock drawing test
MMSE: Mini-Mental State Examination
SST: Serial subtraction task
MPT: Multistep performance task
DSRS: Dementia Severity Rating Scale.

## Supplementary Materials

Supplementary Materials Appendix 1: the modified Mini-Cog has been fully described. It includes tool description, three-word registration (all versions of words), two distractors, and three-word recall. It also includes scoring and interpretation guidelines. Appendix 2: this includes the questionnaires that are used to assess acceptability and feasibility of the Mini-Cog and two versions of the modified Mini-Cog. It also includes how scores were calculated and interpreted.
