# Supplementary material for: Modifying the Mini-Cog to Screen for Cognitive Impairment in Nonliterate Individuals
Source: Int J Alzheimers Dis. 2021 Aug 16;2021:5510093. doi: 10.1155/2021/5510093 (PMC8384546; doi:10.1155/2021/5510093)
Supplement: Supplementary Materials — Appendix 1: the modified Mini-Cog has been fully described. It includes tool description, three-word registration (all versions of words), two distractors, and three-word recall. It also includes scoring and interpretation guidelines. Appendix 2: this includes the questionnaires that are used to assess acceptability and feasibility of the Mini-Cog and two versions of the modified Mini-Cog. It also includes how scores were calculated and interpreted. [file 5510093.f1.docx]

**Appendix 1**

**Modified Mini Cog (MMC)**

*Instructions, administration and scoring of the modified mini cog (MMC) test*

ID: ________________________________ Date: _______________________

**Step 1: Three-word registration**

Look directly at person and say, “please listen carefully. I am going to say three words that I want you ***to repeat back to me now and try to remember***. The words are (select any list of words from the versions below). Please say them for me now.” If the person is unable to repeat the words after three attempts, move on to step 2 (mathematical calculation).

The following word lists have been used in one or more clinical studies (Borson et al., 2000, 2003, 2006, Lessig et al., 2008). For repeated administrations, ***use of an alternative word list*** is recommended.

| Version 1 | Version 2 | Version 3 | Version 4 | Version 5 | Version 6 |
| --- | --- | --- | --- | --- | --- |
| Banana  Sunrise  Chair | Leader  Season  Table | Village  Kitchen  Baby | River  Nation  Finger | Captain  Garden  Picture | Daughter  Heaven  Mountain |

**Step 2: Distractors**

1. **Serial Subtraction Task (SST)**

You have 20 dollars (or rupees, or typical currency of the country). As you mentioned, you would like to drink tea every morning or go for groceries everyday or (any common task that an individual does and spends money everyday or every week or every month). Let us suppose that you spend 3 dollars for ----- (the specified task every time). How much will be left with you if you spend 3 dollars first day from your 20 dollars? If you spend another 3 dollars the next day again, how much will you have left?  …… Continue up to 5 times (the final remaining money should be ***down to 5 dollars at the end of 5 purchase subtractions***). ***Score 2 if all 5 subtractions are correct. Otherwise score 0.*** Move to another step if the calculation is not completed ***within three minutes***. This can be used even for clients with vision calculation.

After each subtraction: 1^st^_____ 2^nd^ ______3^rd^______4^th^______5^th^______(final number)

**OR**

1. **Multistep Performance Task (MPT)**

Take this paper on your less affected or dominant hand (the evaluator gives a piece of any sized square or rectangle shaped paper to the patient/client from his/her one hand), convert it into this shape (evaluator shows a triangular shaped paper by his/her another hand) by tearing or using scissor and then place it on the floor/table. Evaluator gives complete instruction at once. Make sure that he/she understood what the triangle is (there must be three corners or tips and three sides, disregard to degree of angle or length of any side) and how to do the whole task. Then the patient/client has to complete all ***three steps*** (receiving, shaping and placing) without interruption. For ***full score i.e. 2,*** patient/client has to take the paper from the evaluator’s hand, convert it into triangle shape and place it on the floor/table. Otherwise, the score is 0. Move to step 3 if the task is not completed ***within three minutes***. This task can be used even for clients with hearing impairment.

**Step 3: Three-word recall**

Ask individual to recall the ***three words you stated in step 1.*** Say: “What were the three words I asked you to remember?” Record the word list version number and the person’s answers below.

Selected word list version: ____Patient/client’s answer: __________ _________ _________

Scoring and interpretation

| 3-word recall: __________ (0 to 3 points) | 1 point for each word spontaneously recalled without cueing |
| --- | --- |
| Score of SST: ___________  (0 or 2 points)  **OR**  Score of MPT: ___________  (0 or 2 points) | If all five subtractions correctly made and final number is 5, then, score 2, otherwise 0.  **OR**  If all three steps (receiving, shaping and placing) correctly done, score = 2, otherwise 0. |
| **Total score:**  3-word recall + Score of SST: _________  (0 to 5 points)  **OR**  3-word recall + Score of MPT: __________  (0 to 5 points) | **Reference cutoff points**: A cut point of < 3 on the Mini-Cog has been validated for dementia screening, but many individuals with clinically meaningful cognitive impairment will score higher. When greater sensitivity is desired, a cutoff point of < 4 may be used. |

____________________

Assessor (sign and date)

**Appendix 2**

**Questionnaires to assess acceptability and feasibility of Mini-Cog and two versions of a Modified Mini-Cog**

| **Domains** | **Questionnaires** | **Yes: 1, No: 0** |
| --- | --- | --- |
| **Registration of three words** | 1. Were you able to understand those three words told to you? |  |
|  | 1. Were those three words familiar to you? |  |
|  | 1. Were those three words simple to name and remember for you? |  |
| **Serial subtraction task as a distractor**  **OR**  **Multistep performance task as a distractor**  **OR**  **Clock drawing test as a distractor** | 1. Were you able to understand the mathematical calculation as told to you? |  |
|  | 1. Do you do these sorts of mathematical calculations in your daily life? |  |
|  | 1. Were you able to understand this type of mathematical calculation question? |  |
|  | 1. Were you able to understand the multistep task I asked you to do? |  |
|  | 1. Do you do these sorts of multistep task performance in your daily life? |  |
|  | 1. Were you able to understand this type of multistep task performance question? |  |
|  | 1. Are you able to write and read? |  |
|  | 1. Are you able to draw and read a clock? |  |
|  | 1. Are you able to understand this clock-drawing question? |  |
| **Calculation of the score:** Registration score + Score on distractor task  **Total score:** 0-6  **Interpretation:** Higher score indicates greater acceptability and feasibility. | | **Total:** |

____________________

Assessor (sign and date)
